# An Analysis of Employment for Persons with Intellectual and Mental Disabilities in Japanese Labor Transition Support Offices

**DOI:** 10.1192/j.eurpsy.2025.1831

**Published:** 2025-08-26

**Authors:** Y. Katayama, A. Nakao, A. Sasaki, Y. Kadoshita, F. Tomita, A. Ozawa

**Affiliations:** 1 NAGANO UNIVERSITY, Nagano; 2 Decentwork-lab; 3 J.F.Oberlin University, Tokyo; 4 KYOTO UNIVERSITY OF EDUCATION, Kyoto; 5 SAITAMA PREFECTUAL UNIVERSITY, Saitama; 6 University of Tsukuba, Tokyo, Japan

## Abstract

**Introduction:**

In Japan, the Comprehensive Support for Persons with Disabilities Act, which unifies the three disabilities of physical, intellectual, and mental health, came into effect in 2006, and labor transition support offices were institutionalized. The labor transition support business office is a business in the welfare sector that places persons with disabilities in general companies for a period of two years.

- In addition, Japan has the Law for Employment Promotion of Persons with Disabilities in the labor field, which imposes an employment rate for persons with disabilities on companies.
- As of April 2024, the employment rate of persons with disabilities in companies is 2.5%.

**Objectives:**

In order to achieve employment for as many people with disabilities as possible, we analyzed two groups of people with and without workplace retention made by disability at labor transition support offices. This is a secondary data analysis of a national survey conducted in 2022.

**Methods:**

The survey instrument included basic attributes of respondents and establishments, as well as the number of people who found employment in general companies in FY 2019 and their workplace retention status, whether or not employment assistance was provided to people with employment difficulties, and the state of practice of approaches to employment assistance. All statistical analyses were conducted using SPSS. Ethical review was conducted with the approval of the Ethical Review Committee of Nagano University (2021-006).

**Results:**

The types of disabilities of the establishments (n=1321) indicated 19 establishments with physical disabilities, 286 establishments with intellectual disabilities, 250 establishments with mental disabilities, 52 establishments with developmental disabilities, 495 establishments with no specific disability, and others.

In this analysis, from the above-mentioned establishments with intellectual disabilities (n286) and establishments with mental disabilities (n250), we divided them into two groups: those with intellectual disabilities (n181) and those with mental disabilities (n181) that discharged employed persons in FY2019.

It was found that the labor transition support offices that were placing people in employment with mental disabilities as their primary disability were accepting those with fewer weeks of use of the offices and with more difficulties in employment.

**Conclusions:**

It became clear that even though the legal system unifies the three disabilities, the approach differs depending on the characteristics of the disability. Furthermore, it was also found that there are some offices that are able to place users in employment even if they have difficulties in finding employment. This suggests that there is a possibility that labor transition support offices, which are unable to produce job seekers, will be able to produce job seekers in the future.

**Disclosure of Interest:**

None Declared

